# Typologies of duocentric networks among low-income newlywed couples

**DOI:** 10.1017/nws.2023.16

**Published:** 2023-08-25

**Authors:** David P. Kennedy, Thomas N. Bradbury, Benjamin R. Karney

**Affiliations:** 1 RAND Corporation, Santa Monica, CA, USA; 2 University of California, Los Angeles, CA, USA

**Keywords:** Duocentric networks, personal networks, marriage, couples, cluster analysis, cognitive networks

## Abstract

The social networks surrounding intimate couples provide them with bonding and bridging social capital and have been theorized to be associated with their well-being and relationship quality. These networks are multidimensional, featuring compositional (e.g., the proportion of family members vs. friends) and structural characteristics (e.g., density, degree of overlap between spouses’ networks). Most previous studies of couple networks are based on partners’ global ratings of their network characteristics or network data collected from one member of the dyad. This study presents the analysis of “duocentric networks” or the combined personal networks of both members of a couple, collected from 207 mixed-sex newlywed couples living in low-income neighborhoods of Harris County, TX. We conducted a pattern-centric analysis of compositional and structural features to identify distinct types of couple networks. We identified five qualitatively distinct network types (wife family-focused, husband family-focused, shared friends, wife friend-focused, and extremely disconnected). Couples’ network types were associated with the quality of the relationships between couples and their network contacts (e.g., emotional support) but not with the quality of the couples’ relationship with each other. We argue that duocentric networks provide appropriate data for measuring bonding and bridging capital in couple networks.

## 1. Introduction

Social ties benefit intimate couples in myriad ways, providing advice and emotional support, as well as more tangible resources, such as childcare and financial support (Cornwell, 2012; Haggerty et al., 2022; Wellman & Wortley, 1989; Widmer, 2004). Yet social ties can also harm intimate partnerships by placing demands on couples’ time and energy or by draining financial resources (Bryant & Conger, 1999). In addition to their direct influence, social ties provide a key source of behavioral norms (Coleman, 1988; Widmer, 2004) that inform partners’ decision-making regarding childbearing and child rearing (Widmer et al., 2006), household division of labor (Bott, 1957), as well as communication and problem resolution (Felmlee, 2001). The social environment of couples appears to affect their intimate relationships as well, having been associated with relationship satisfaction (Cotton et al., 1993), infidelity (Treas & Giesen, 2000), and dissolution (Booth et al., 1991; Burger & Milardo, 1995; Felmlee, 2003; Fiori et al., 2020; McDermott et al., 2013). To date, associations between characteristics of shared couple networks and relationship outcomes have been inconsistent, with some studies showing a link (Fiori et al., 2020) but not others (Bryant & Conger, 1999).

Social capital theory provides a framework for understanding the diverse and sometimes conflicting impacts of social relationships on intimate relationships and families (Aeby et al., 2014; Fiori et al., 2020; Fiori et al., 2018; Widmer, 2006). Merging the social networks of two individuals into a joint couple network is often considered beneficial because a couple has access to the combined social resources of two people (Acock & Demo, 1994; Curran et al., 2003; Kalmijn, 2003). However, the same dynamics that produce positive outcomes can also trigger negative consequences (Kawachi et al., 2008; Portes, 1998). A key to understanding these consequences is differentiating between *bonding* and *bridging* social capital (Coleman et al., 2022; Kawachi et al., 2008; Widmer, 2006). Bonding social capital refers to close, kin-centered relationships that offer a high level of cohesion from a densely connected and homogenous social environment (Coleman, 1988). Networks high in bonding social capital provide high levels of support and a sense of belonging to members of a connected community, but may also trigger interference, control, excessive demands, expectations of conformity, and an emphasis on in-group solidarity and exclusion of out groups (Johnson & Milardo, 1984; Kawachi et al., 2008; Milardo et al., 2014). In contrast, bridging capital results from a diversity of connections across different groups, which can provide access to novel financial, emotional, or informational resources (Burt, 1992; Burt, 2004; Granovetter, 1973).

Understanding how social capital impacts couples’ well-being and development in the context of key life events—such as marriage, childbearing, retirement, and divorce—requires precise and reliable measurements of bonding and bridging capital in couple social networks, i.e., the people with whom partners have regular interaction, and the relationships among these people (Antonucci et al., 2010). While bonding and bridging capital are relevant for all couples (Kim et al., 2006), distinguishing between bridging and bonding capital is especially useful for investigating the impact of social ties on members of economically disadvantaged communities (Kawachi et al., 2008). Bonding capital can be an important survival mechanism to compensate for low financial capital (e.g., wealth, income) and/or low human capital (e.g., education). However, high bonding capital often comes at a cost: persistent demands for social support in the social networks of those in high-poverty communities and the expectations of reciprocal support (Mitchell & LaGory, 2002). Bridging social capital, on the other hand, can help provide opportunities for upward mobility by increasing access to new resources (Granovetter, 1973; Johnson et al., 2011).

Although social capital has been conceptualized and measured in different ways (Kawachi et al., 2008; Lakon et al., 2008), social network analysis provides a useful framework for mapping the flow of social investments through social systems of relationships and interactions toward recipients of resources (Milardo et al., 2014). Network measures of social capital include functional measures (the content of ties), structural measures (the interconnections among all members of a network), and positional measures (the number of structurally non-redundant ties available to one member of a network) (Lakon et al., 2008). Many traditional measures of egocentric and sociometric networks can be employed to operationalize bonding and bridging capital (Borgatti et al., 1998; Lakon et al., 2008), such as network density, homophily, centralization, etc. (Borgatti et al., 1998). Other network-based measures of social capital have been explicitly developed to operationalize social capital, for example *constraint* (Burt, 1992), which measures the extent that an individual in a network has access to high bridging capital through many non-redundant network contacts (Everett & Borgatti, 2020).

Generating these measures of bonding and bridging capital for couple networks requires enumeration of members of this network and precise measurements of characteristics of these network members and the ties among them. Since the seminal work of Bott (1957), relationship scientists and personal network researchers have investigated the structure of couples’ shared social networks (Perry et al., 2018; Widmer, 2004). Yet despite decades of investigations, approaches to measuring those networks remain inconsistent. Most prior studies have measured network characteristics with imprecise global assessments of network characteristics—e.g., counts of types of network members or ratings of the characteristics of networks in general—or conducted personal network interviews with only one member of the couple (e.g., Cornwell, 2012; Felmlee, 2001; Sprecher & Felmlee, 2000). Addressing couple networks from the perspective of one member of the couple is problematic because couples, by definition, consist of two people (a dyad), each at the center of their own egocentric network that intersects with their partner’s egocentric network to varying degrees.

A small number of studies have followed the lead of Bott and directly measured couple networks by first measuring the personal networks of both members of the couple and then merging them together into one “duocentric” configuration (Brands, 2013; Coromina et al., 2008; Jackson et al., 2016; Julien et al., 1999; Kennedy et al., 2015; Stein et al., 1992). Combining each partner’s individual perceptions of their personal networks into a measure of their shared social environment through a Cognitive Social Structure (CSS) approach (Brands, 2013; Krackhardt, 1987) retains the separate perspectives of the members of the couple while also accounting for their non-independence (Kenny et al., 2006). Following Krackhardt (1987), the couples represent two “slices” of a CSS transformed into a Consensus Structure based on a threshold function (e.g., a tie between alter *i* and alter *j* exists if both spouses perceive that a tie between *i* and *j* exists). Measurements produced from duocentric data enable testing of the range of theories about the relevance of bonding and bridging capital to intimate couple development at multiple levels of analysis (e.g., at the overall duocentric level or at the individual spouse level nested within the duocentric level).

A challenge to testing theories with duocentric network data is that, much like personal network data, it can be used to generate many different indices that are distinct, yet highly correlated. Data reduction techniques, such as cluster analysis, enable summary of these variables into a single index (Antonucci et al., 2010; McCarty et al., 2019). Using this “pattern-centric” or “configural” approach has been found to improve explanatory power in models that include network types compared to a traditional linear approach that included individual network measures as separate variables (Stein et al., 1992). This pattern identification approach has been used in personal network studies across a variety of populations (Bidart et al., 2018; Friedman & Kennedy, 2021; Giannella & Fischer, 2016; Green et al., 2012; Litwin et al., 2020; Maya-Jariego, 2021; Vacca, 2019) and has a long history of use in research on family personal networks (Friedman & Kennedy, 2021; Ladd & McCrady, 2016; Park et al., 2015; Santini et al., 2015).

To date, only a handful of studies have used a pattern-centric approach to classify couple networks into “conjugal types” using reports from both members of a couple (Fiori et al., 2017; Stein et al., 1992; Widmer et al., 2004). Stein et al. (1992) was the only study to analyze duocentric data, generated from separate interviews with 47 White spouses who had been married for more than 5 years. Although the study reports collecting data on interconnections among network members, only counts of shared and separate friends and family members were analyzed to produce discrete clusters. The study did not test for associations between network types and network relationship quality. The studies by Widmer et al. (2004) and Fiori et al. (2017) identified conjugal types based on separate responses to survey items by both members of the couple, but did not measure duocentric characteristics directly. Although both studies found associations between network types and conjugal quality, many of the survey items used to identify cluster types bundled together aspects of network structure, composition, and/or relationship quality between egos and their alters. Therefore, clusters produced in these studies are results of partners’ general perceptions of their network structure, network composition, and network quality that cannot be disaggregated to test for associations between network composition or structure with network relationship quality.

The primary aim of the current study is to classify duocentric networks of newlywed couples empirically based on measures of bridging and bonding social capital and to test for the association between identified types and conjugal network quality. The current study is the first to identify conjugal types among newlywed couples and only the second to assess the duocentric characteristics of newlywed couples (Jackson et al., 2014; Kennedy et al., 2015). Because divorce is common in the early years of marriage (Bramlett & Mosher, 2002; Copen et al., 2012; Raley & Sweeney, 2020), an advantage of studying newlywed couples over couples in established relationships is that couples who may go on to divorce have not yet exited the population. Given the hypothesized association between couple network characteristics and relationship outcomes (Copen et al., 2012), sampling established relationships may provide misleading results. Thus, studying the social networks of couples in early stages of their marriage is essential to understanding how bonding and bridging capital intersect in couple networks and affect how couples cope with stress that may contribute to relationship dissolution, such as living in high-poverty environments (Lyons et al., 1998).

The previous theoretical and empirical literature on couple networks suggests several hypotheses that we address in our analyses. First, we hypothesized that we would find qualitatively distinct types of newlywed couple networks distinguished by duocentric measures of bonding and bridging capital. Measures of bonding capital include relative amounts of family, proportion of network members shared by spouses, and the amount of connections among network members overall. Measures of bridging capital include relative amounts of non-kin/friends, network contacts primarily tied to one spouse or the other, and the amount of disconnected subgroups (e.g., husband family, wife family, etc.). Second, based on previous studies, we anticipated that relationship quality between couples and the members of their network would differ across these network types. Informed by social capital theory (Coleman, 1988), we hypothesized that different types of duocentric networks with different constellations of bonding and bridging connections among members would impact the social resources spouses receive from their social ties as their separate egocentric networks merge into one duocentric network centered around the married dyad. The impact of networks on couples is multidimensional, including close and positive relationships, support received, frequency of interaction, and either approval of the marital relationship or interference in the relationship. Finally, we anticipated that network types associated with higher quality relationships with network members would also be associated with higher perceived quality of the relationship between the spouses themselves. We hypothesize that couples who have an immediate social environment that balances both bonding and bridging capital, is cohesive, supportive, with many positive and supportive relationships and includes a balance of friends and family of both spouses, are more likely to perceive that their spousal relationship is positive and satisfying.

## 2. Design and methods

### 2.1. Sampling strategy and participants

The data for this paper were collected as part of a broader longitudinal study of the trajectory of newlywed marriage among couples living in low-income neighborhoods. Newlywed couples were identified via marriage license records obtained from the Harris County, Texas Recorder’s Office between 2014 and 2015. Addresses were matched with census data to identify applicants living in high-poverty communities, defined as census block groups for which no less than 30% of the households were categorized as living below poverty (U.S. Census Bureau, 2008). A total of 4,916 couples were identified through this marriage licenses matching process and 3,759 were contacted first by mail and then by telephone. Of those contacted, 1,157 agreed to be screened for eligibility in person or over the phone. To be eligible, partners had to be in their first marriage, living together (i.e., neither partner could be deployed or incarcerated), and above 18 years of age. Those who were eligible and provided consent were included in the study. Of those screened, 506 couples were determined to be eligible, of which 401 agreed to participate in the study and 231 actually participated in data collection. Among the couples who provided any data, 226 provided egocentric data from husbands and wives (97%) and 207 (89%) provided sufficient egocentric data from each spouse to facilitate categorization (i.e., at least 20 alters were named by each spouse and complete alter-alter tie evaluations could be made).

### 2.2. Data collection strategy: personal networks

Couples were visited in their homes by two trained interviewers who interviewed spouses in separate rooms. The interview content and procedures were fully explained, and informed consent was obtained from each spouse. Husbands and wives were interviewed separately using EgoWeb 2.0, open-source computer-aided interviewing software customized for network data collection (http://egoweb.info). Spouse network interviews averaged 40 minutes each. Respondents gave their answers to questions verbally, and interviewers recorded their responses on laptop computers. Following established procedures for conducting personal network interviews (Campbell & Lee, 1991; Crossley et al., 2015; McCarty, 2002; McCarty et al., 1997; McCarty et al., 2019; Perry et al., 2018), the personal network interviews were divided into three sections: a name generator to identify lists of alters, name interpreter questions to generate responses about alter characteristics and ego-alter relationships, and questions about the relationship between each unique pair of network alters.

### 2.3. Alter name generation

Similar to the approach used in a study of the duocentric networks of newlywed couples in Los Angeles (Kennedy et al., 2015), each spouse (the “ego”) was prompted by a generic name generator for a fixed number of network alters coupled with non-specific probing. Each spouse was asked to name their spouse and 24 additional alters for a total of 25 alters, which is large enough to counteract bias that is introduced with a single, specific name generator while also short enough to limit respondent burden (Bidart & Charbonneau, 2011; Brewer et al., 1999; Marin & Hampton, 2007; McCarty et al., 2007). The target number of 25 alters was also identified as the point at which structural and compositional characteristics stabilized in the study of duocentric networks of newlywed couples in Los Angeles (which elicited 40 alters from each spouse) (Kennedy et al., 2015). See Appendix A of the Supplementary Materials for an extended discussion of our decision to prompt respondents to name 25 alters. The exact wording of the name generator used in this study is as follows:

“I’d like you to name 25 people that you know and who know you. Here’s the kind of person we are hoping you will name: first, they have to be adults, aged 18 years old or older—do not give me the names of children under age 18; second, these should be people you have had contact with sometime during the past year or so—either face-to-face, by phone, mail, or email; third, these do not have to be people you like, just people you know and who know you. Let’s start by naming your spouse, and after that you can name any adults you know no matter who they are or where they live. Please give us their first and last names. Remember, all of the information you give us is confidential.”

To facilitate matching unique alters named across spouses, we asked respondents to give first and last names rather than first names only or initials as is customary in personal network interviews. If respondents did not want to give last names or did not remember last names, they were given the option to give the first few initials of the last name or a nickname or description of the person. The procedures for collecting and storing alter name data were developed with the guidance of the authors’ Human Subjects Protection Committee.

### 2.4. Collection of raw network composition and structure data

For alter assessments, we asked respondents if the alters were their own or their spouse’s family member, their friend or their spouse’s friend, a coworker, neighbor, etc. We allowed for more than one of these options to be selected for any particular alter (e.g., one alter could be rated by a respondent as their own and their spouse’s friend). For relatives, we asked how the alter was specifically related to the respondent (e.g., mother/father, brother/sister). We also asked a series of ego-alter relationship questions, including how well they knew the alter (“Very well,” “Pretty Well,” or “Not Well”), the quality of the relationship (e.g., frequency of contact, receipt of different types of support), and demographic characteristics of the alter (e.g., marital status, if they had children under 18, employment status).

To measure network structure, we asked respondents to assess the relationship between each unique pair of network alters with the following prompt:

“Going back to the list of 25 people that you mentioned earlier, I am going to ask you about pairs of these people and whether they have had contact with each other sometime during the past year or so—either face-to-face, by phone, or e-mail. For each pair, I want to know if the two people have had any contact.”

If the two alters had contact with each other in the past year, we also asked how well they knew each other (“Very Well,” “Pretty Well,” or “Not Well”).

### 2.5. Construction of duocentric networks from separate egocentric networks

We followed procedures recommended for constructing duocentric networks from separate husband and wife personal network assessments (Kennedy et al., 2015), in which the separate husband and wife alter-alter relationship ratings were combined to form each couple’s duocentric network. First, we identified matching alters named by both spouses by comparing first and last names. Potential matches were further examined by comparing the corresponding relationship types identified by each spouse (e.g., a husband’s father and a wife’s father-in-law) as well as answers to demographic questions (e.g., marital status, employment status). Once we had identified matching alters, we created a unique identifier and merged it into the duocentric data set as one person. For alters with data from both spouses, we calculated a set of maximum, minimum, and average responses for the two spouses. In this study, we use maximum values when the ratings provided by each spouse differed. For example, if a husband thought that two alters knew each other “Pretty well” but the wife thought that they knew each other “Very well,” we used the wife’s evaluation for the combined couple network data. Wives and husbands were included as nodes and the ratings of their own ties to each alter were included as edges. Because spouses were not asked to rate how well they knew each other in the initial interview, the edge between each spouse was set to the maximum tie strength value.

### 2.6. Duocentric structure: density, components, constraint

Once couple networks were constructed, we developed measures of duocentric network structure and composition for each couple’s network. We produced two types of measures: (1) measures of duocentric couple network composition and structure likely to be indicators of bonding and bridging capital that we hypothesized would be key characteristics of discrete types of duocentric network types and, (2) measures that would be significantly associated with these network types based on our expectations of the effects of combinations of bonding and bridging capital. We calculated measures of network composition that have been hypothesized to impact couple relationships, such as the proportion of the networks made up of family or friends and the amount of these types of network members that were shared by spouses. Measuring relative amounts of family and friends is an important indicator of bonding/bridging capital because couple networks high in bonding capital have been theorized to be kin-centered rather than friendship centered. We also calculated proportions of shared network members, for example those named by both spouses in their own egocentric networks, to construct measures of network overlap. High network overlap has been theorized as a key indicator of bonding capital. We constructed network structural measures based on the raw responses to the relationship evaluation questions provided by the spouses.

After constructing duocentric networks for each couple by identifying the same network members named by both spouses, we calculated a measure of *network size* by counting the number of uniquely named alters. Because each spouse was asked to name their spouse and a maximum of 24 additional alters, the theoretical maximum duocentric network size was 50 for a network, which included the spouses and 24 unique alters named by each spouse, while the theoretical minimum was 25 if each spouse named the same exact list of alters. To measure overall network connectivity, a key measure of bonding capital, we calculated the overall duocentric network *density*. To capture connectivity among duocentric alters who were likely to interact and communicate, we calculated density based on a duocentric network matrix consisting of alter-alter ties that were rated as either “Pretty Well” or “Very Well” (and set those with either no contact or knowing each other “Not Well” to 0). Based on this same tie definition, we calculated duocentric *components*, which is a count of all disconnected groups (sets of network members with either a direct or indirect tie). Measuring disconnected groups within the duocentric network is an important assessment of “bridging” capital because it indicates that the spouse dyad acts as a bridge between otherwise disconnected network members. Following recommendations for calculating components for duocentric networks (Kennedy et al., 2015), both the density and components measures were derived from the matrix of alter-alter ties only and excluded ties between the spouses and the alters. By definition, the list of alters was populated by names of people provided by the spouses as their network contacts. Therefore, with spouse alter ties included in the duocentric networks, each tie is either directly or indirectly connected to each other through a shared connection to one of the spouses and all duocentric component calculations will produce one component. Excluding spouses thus provides more precise insight into the structure of their shared duocentric networks, including cohesiveness (low number of components) and fracturing (large number of components).

### 2.7. Network composition: family, friends

We calculated measures of family composition by counting the number of alters identified as being either a member of the husband’s or wife’s family and dividing this number by the network size. For each alter named in the separate egocentric interview, each spouse was asked “How do you know ?” Respondents were read a list of example relationship types, including a family member, a member of their spouse’s family, their friend, their spouse’s friend, their coworker, neighbor, former romantic partner, service provider, or something else. Alters identified as either a family member by the husband and/or an in-law by the wife were classified as a member of the husband’s family. We divided the count of these alters by the duocentric network size to calculate the proportion of the network that was *husband family* or *wife family*. We classified alters as a husband’s friend if the husband identified the alter as his friend and/or the wife identified the same alter as her spouse’s friend. We divided the count of these alters by the total network size to produce proportion of *husband friend* and similarly calculated as the proportion of *wife friend*.

### 2.8. Network overlap: shared friends, shared nomination

After alter names were matched across each spouse’s egocentric interview, we classified those who were named by both spouses and divided the total of these alters by duocentric network size to produce proportion of *both nominated*. For alters named by both spouses as their own friend or by one spouse as both their friend and their spouse’s friend, we classified the alter as a shared friend. We divided the count of these alters by the network size to produce the proportion of *shared friends*.

### 2.9. Measures: spouse alter nominee density

In addition to measuring characteristics of the network as a whole, we calculated sub-network measures for spouse nominees. Previous couple network classification studies identified couple networks that were primarily “patricentric” (primarily connected to the husband) or “matricentric” (primarily connected to the wife) (Widmer et al., 2004). To determine if bonding capital was primarily driven by connections to one spouse or the other, we first identified those nominated by the husbands and calculated *husband-nominated density* (density among only those network members) and similarly calculated *wife-nominated density.*

### 2.10. Measures: spouse constraint

To test if network types were associated with how much duocentric networks expose couples to diverse or limited social opportunities and, therefore, different levels of bridging social capital, we measured *constraint* based on the measure defined by Burt (2004) for each spouse within their shared network. Network constraint is a summary measure of how connected one network member is to network members who are also directly or indirectly connected to each other and, therefore, have redundant structural positions (Burt, 1992, 2004; Burt et al., 1998; Everett & Borgatti, 2020). Individuals with low constraint have higher exposure to novel social experiences, resources, and information flows and, therefore, higher bridging social capital. We calculated *wife constraint* and *husband constraint* for the spouses within the same duocentric networks based on strongest ties only (ties who know each other “Very Well”). See Appendix A of the Supplementary Materials for additional discussion of the constraint measure, in particular the tests of association between constraint and duocentric clusters.

### 2.11. Measures: network relationship characteristics

To test if different network types with different configurations of bonding and bridging capital were associated with network relationship quality, we constructed a series of network relationship quality composition measures. For each alter named by the spouses, they were asked a series of questions that were designed to measure different elements of their relationship with these network contacts. We calculated proportions of types of alters by totaling the number of alters with a given response and dividing by the duocentric network size. Spouses rated how well they knew the alter (“very well,” “pretty well,” or “not well”). We calculated variables measuring the proportion of alters spouses said that they *know very well*, *know pretty well*, and *know not well*. Spouses also rated their relationship with each alter they named: “good,” “neutral,” and “bad.” We totaled the number of alters receiving each rating and divided that total by the duocentric network size to produce *relationship good*, *relationship neutral*, and *relationship bad* proportion measures. Spouses also identified the alters from whom they received “concrete support, such as money, transportation, food, or anything else” and “emotional support, like encouragement, or someone to talk to about their feelings.” We calculated counts of each of these responses and calculated proportions of *tangible support received* and *emotional support received*. Spouses were also asked to report how the alters they named felt about their marriage: approve, disapprove, or had no opinion. We totaled alters receiving each response and calculated proportions of alters who *approve*, *disapprove*, and had *no opinion*. Spouses rated how frequently they interacted with each of the alters they named in the past year, both face-to-face and electronically (email, phone, text, etc.): “every day,” a “few times a week,” “once a week,” etc. These responses were converted to total days and these responses were summed and divided by the duocentric network size to produce average measures of frequency of *face-to-face contact* and *virtual contact*. See Appendix A of the Supplementary Materials for additional discussion of relationship strength measures.

### 2.12. Measures: couple relationship satisfaction

Spouses were asked to rate their satisfaction with their marriage. Husband and wife relationship satisfaction, conceptualized as spouses’ global sentiment toward the relationship, was assessed using ten items from the Couple Satisfaction Index (CSI-16; Funk & Rogge, 2007), with higher scores indicating higher levels of satisfaction. The items assessed global satisfaction (e.g., “My relationship with my partner makes me happy”) and were rated on a 6-point scale. We calculated *husband satisfaction* based on the husband’s responses and *wife satisfaction* based on the wife’s responses. In addition to exploring associations between network types and separate spouse satisfaction, we also tested for an association between network type and combined spousal satisfaction to account for the non-independence of spouse relationship satisfaction and its likely non-independent association with duocentric network characteristics. To generate this combined satisfaction measure, we followed the approach of studies that have tested the association between couple typologies and relationship satisfaction (Ladd & McCrady, 2016; Lavee & Olson, 1993). We used this approach to calculate a couple score that measured the satisfaction of the spouses with the following formula with *k* = .5:

$$C=\frac{h+w}{2}-k\frac{\left|h-w\right|}{2}\;\text{where:}\;1\leq k\leq 0$$

The formula weights the average relationship satisfaction between the couples (h+w/2) by the bsolute value of the difference between the spouses’ satisfaction scores (|h−w|/2) multiplied by a constant that is set to .5. The constant k ranges from 0 (which results in the mean relationship score) to 1 (which results in the lower relationship score).

### 2.13. Analysis: identifying couple network typology

We classified the duocentric networks of the couples in a two-staged cluster analysis approach using the statistical package R version 3.62, hierarchical and *k*-means clustering. We selected 10 duocentric variables that were informed by our hypotheses and operationalized key dimensions of duocentric networks: two measures of network structure (duocentric network density and components), four measures of network composition (proportions of wife family, husband family, wife friend, husband friend), two measures of duocentric overlap (shared friends, shared nominations), and two measures of subgroup structure (wife-nominated alter density, husband-nominated alter density). After generating a distance matrix of standardized transformations of these measures, we first applied the hierarchical clustering procedure using Ward’s error sum of squares variance method with the “hclust” function in the R package “stats” (Murtagh & Legendre, 2014). We also ran the “NbClust” function in the R package “NbClust” with the “k-means” method, which produces 30 indexes of cluster fit on a range of numbers of clusters and examined the distribution. After examining a histogram of cluster by number of best-fit diagnostics, we conducted several other diagnostics to identify the best number of clusters to analyze. For example, we produced dendrograms to visually display the level of closeness when groups of observations split into separate clusters and elbow plots of the total within-cluster sum of squares to visually identify noticeable differences between number of clusters. We used these measures of cluster fit to determine the significant peak in the number of clusters that corresponded with the highest number of best fits among all the indices.

Finally, we compared the deductive classification results with an inductive comparison of the characteristics of the clusters to determine an optimal number of meaningful clusters. We evaluated several of the best cluster partitions identified by the various clustering evaluation packages for meaningfulness by comparing within-cluster means of the variables included in the cluster analysis to the overall means. Following an approach to interpreting couple network classification based on global assessments of network characteristics (Fiori et al., 2017), we first standardized the means within clusters to aid in comparison of variables having different scales (e.g., counts and proportions). We produced *t*-scores of means of each variable within each cluster by subtracting the within-cluster means from the overall mean and dividing by the overall standard deviation and standardizing these measures to a mean of 100 and a standard deviation of 10. We plotted *t*-scores by cluster membership and examined the pattern of extreme values to interpret the characteristics of the networks that best described the cluster. This inductive analysis process guided the selection of clusters and the naming and description of the clusters. Once we identified the clusters, we tested for association between duocentric network type and the measures of duocentric structure and composition included in the cluster analysis. We examined the ANOVA results to identify which variables were significantly associated with variance between clusters on the criterion network measures.

### 2.14. Analysis: logistic regression

Once we identified duocentric network types, we constructed models to test for significant associations between independent variables and cluster membership. We tested associations between independent variables and cluster membership in two stages. First, for each cluster group, we constructed bivariate logistic regression models using the “glm” function in the R package “stats” with a dependent variable equal to 1 if the couple was a member of the cluster group or equal to 0 if a member of another group. We examined odds ratios (OR) and *p*-values from these models. Next, we constructed multinomial models for the same set of independent variables using the “multinom” function in the R package “nnet.” These models tested which pairs of clusters significantly differed in their association with the independent variable. We examined relative risk ratios (rrr) and *p*-values for these models. Independent variables included couple demographic measures (spouse age, education level, race/ethnicity), couple relationship measures (satisfaction and relationship length), duocentric constraint, duocentric relationship qualities (knowing very well, having a good relationship, tangible support received, emotional support received, approving marriage, frequency of contact face to face, and frequency of contact virtually). See Appendix A of the Supplementary Materials for additional discussion of model selection.

## 3. Results

### 3.1. Descriptive statistics and cluster definitions

Table 1 provides descriptive statistics on the demographics of the 207 couples providing complete network interviews. These couples were predominantly from low SES backgrounds, with low mean annual income ($46,200, SD = $34,900) and mean years of education (women = 14.4, SD = 3.3; men = 13.7, SD = 3.4). The couples’ race/ethnicity was primarily non-White, with both spouses reporting being either Latino/Hispanic (44.9%), African American (29.9%), or Asian/Pacific Islander/Native Hawaiian (1.5%). Both spouses reported being White in 13 of the couples (6.3%) with the remaining couples having spouses with mixed race/ethnicity (17.4%). The mean length of marriage for the couples at baseline was 4.82 months (SD = 4.5). Men’s mean age was 29.3 years old (SD = 7.3) and women’s mean age was 27.6 years old (SD = 6.9). Couples had a mean of 2.8 children (SD = 1.9) in the household. Table 2 provides descriptive statistics (means and standard deviations) of the duocentric networks, including duocentric structure, composition, overlap, constraint, subgroup density measures, and descriptive statistics of the duocentric relationship qualities.

**Figure 1.**
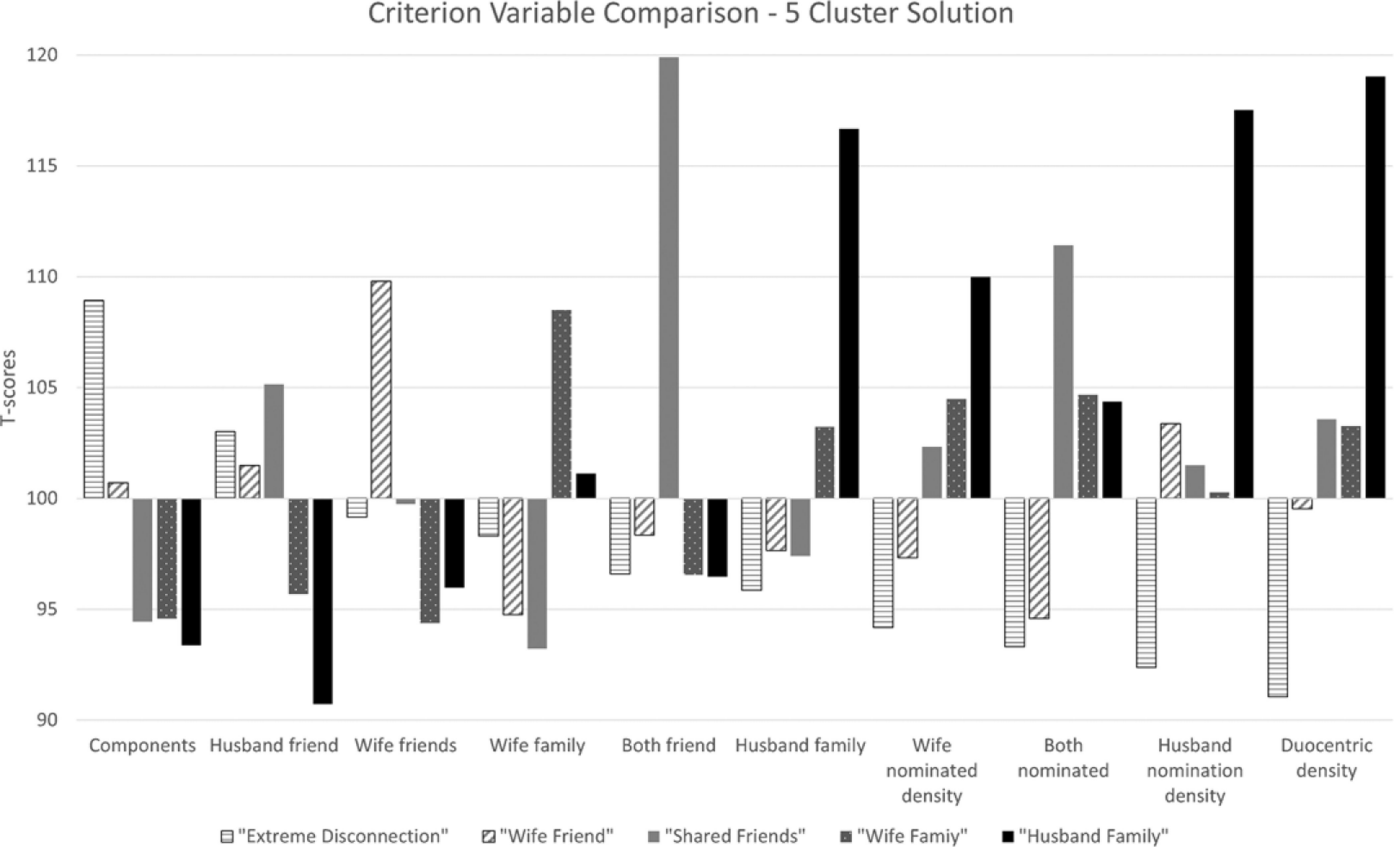
Standardized mean scores (*t*-scores) for criterion variables by network type 5-cluster solution. Raw measures of criterion variables have been converted to *t*-scores with mean = 100 and standard deviation = 10 in order to standardize the height of the bars to facilitate visual comparison among the variables.

Examination of the baseline cluster analysis diagnostics resulted in the selection of a 5-cluster solution (See Appendix B of the Supplementary Materials for more details of these diagnostic tests). To interpret the resulting clusters, we examined the means within each cluster compared to the overall sample. Each cluster criterion variable is presented in the Figure 1 bar chart for the 5-cluster groups with variables sorted by their *t*-scores, which are standardized averages to facilitate comparison across types of variables so that each variable is transformed to have a mean of 100 and a standard deviation of 10. Table 3 provides the means and *t*-scores of the criterion variables used in the 5-cluster analysis. Table 3 also provides the effect size (eta-squared), test-statistic (*F*-value), and *p*-value of ANOVA tests of association between clusters and criterion network measures. As expected, the criterion variables included in the cluster analysis each had a large effect size and were all significantly associated with cluster membership at the 95% confidence level. Examination of the distribution of these within-cluster means/*t*-scores informed the cluster group labels: (1) “Disconnected” (*n* = 60), due to the lowest density and most fracturing of any of the groups; (2) “Wife Friend” (*n* = 27), due to the relatively high numbers of friends primarily affiliated with the wife; (3) “Shared Friend” (*n* = 27), which is distinguished by relatively high numbers of shared friends and shared nominations; (4) “Wife Family” (*n* = 60), distinguished by relatively high numbers of the wife’s family and high interconnections among those nominated by the wife; and (5) “Husband Family” (*n* = 14), containing a relatively high number of husband family members and high density among those nominated by the husband. Both family groups had relatively high number of shared nominations, low friend nominations, shared and non-shared, and high overall density.

**Figure 2.**
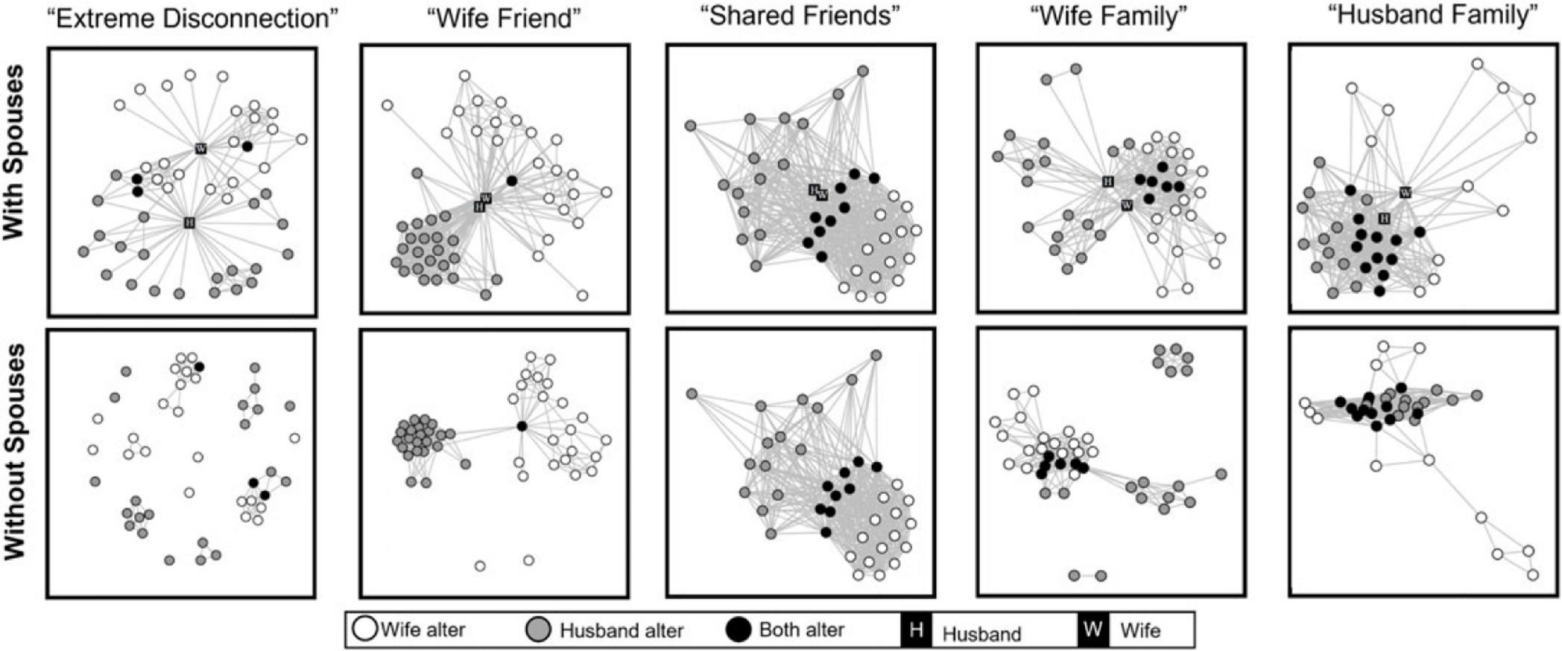
Examples of visualizations of duocentric networks for each of the five cluster types. Example diagrams were chosen to visually illustrate cluster criterion variables described in the manuscript (density, components, etc.). Notes: Nodes represent alters named by wives only (white circles), husbands only (grey circles), or both spouses (black circles). The top row of diagrams includes spouses as nodes represented by black squares (H husband, W wife). Bottom row depicts the same couple networks without the spouses included. The layout of the nodes is generated with the Fruchterman–Reingold force-directed placement algorithm with edges indicating that either spouse indicated two alters knew each other.

Figure 2 presents example visualizations of five different duocentric networks representing each of the five clusters. Each of the diagrams are visualized with the R package igraph using the Fruchterman–Reingold spring embedding layout based on edges defined as alters who know each other. The figures were selected for their illustration of the criterion variables used in the cluster analysis that were key to informing group allocation. The top row provides visualizations of the duocentric networks with spouses included in the diagram and the bottom row shows these same networks with the spouses excluded. Each visualization includes alter nodes identified by which spouse named the alter in the personal network interview (white = wife alter; gray = husband alter) or if the alter was nominated by both spouses (black). The visualizations with spouses removed illustrate the contrasting structural characteristics of the two types of couple networks. The “Extreme Disconnection” example has the lowest density (.13) with spouses included and with a large number of components and isolates (16) when spouses are removed. In contrast, the “Shared Friend” example has the highest density (.62) with spouses and maintains one densely connected component even after the spouse nodes are removed (.58). The “Wife Friend” example has the lowest number of shared spouse nominations (1) and, in the non-spouse graph, mostly splits into two groups nominated by the separate spouses with the exception of two isolates and the shared node, which acts as a bridge between the two groups. The “Wife Family” and “Husband Family” examples have relatively large numbers of shared nominations which are intertwined with a group of wife- or husband-nominated nodes, respectively.

### 3.2. Results: bivariate logistic and multinomial regression

Table 4 presents results of a series of regression models testing for association between cluster membership and measures of couple demographics, couple relationship characteristics, duocentric constraint, and duocentric relationship quality. Each row of Table 4 presents results of a test of a bivariate association between an independent variable and the odds of a couple’s duocentric network belonging to the column cluster (dependent variable = 1) relative to other clusters (dependent variable = 0). Each row presents ORs, 95% confidence intervals, and indicators of *p*-values less than .10, 05, or .01. In addition, Table 4 also indicates which pairs of cluster groups significantly differed in multinomial regression models testing for differences between each pair of clusters on the independent variable. The Race/Ethnicity rows are based on one model with three dummy variables indicating couple race/ethnic characteristics (Black, White, or Other) with Latino as the reference characteristic. Because the “Husband Family” cluster was small (*n* = 14, 6.8%) and had a similar pattern of cluster criterion measures as the “Wife Family” group relative to the mean, we combined these into one “Family” group for logistic/multinomial models.

Several demographic characteristics of couples were associated with cluster membership. Couples with older husbands were more likely to belong to the “Extreme Disconnection” group relative to other groups: for each additional year of age of a husband, couples had 5% increased odds of belonging to this group. These couples had significantly higher husband age than those in the “Shared Friend” group (rrr = 1.08, *p* < .05) and the “Family” group (rrr = 1.09, *p* < .05). Higher education levels of both husbands and wives were associated with membership in the “Shared Friends” group. The odds of being classified into this group increase 2.91 times for each additional year of a husband’s education and 2.13 times for each additional year of a wife’s education. “Shared Friends” couples had significantly higher education than each of the other groups (rrr 1.96–3.29, *p* < .01). Relative to Latino couples, White couples had 8.7 times the odds of membership in the “Shared Friends” group and had significantly higher odds of belonging to this group over the “Family” group (rrr = 11.77, *p* < .001) and the “Extreme Disconnection” group (rrr = 15.91, *p* = .01). Also, relative to Latino couples, those who were not classified as White or Black had 2.58 times the odds of being classified into the “Extreme Disconnection” group and this group was significantly different than the “Family” group (rrr = 2.81, *p* < .05).

For couple relationship characteristics and constraint within their duocentric networks, only constraint was significantly related to couple membership. Odds of belonging to the “Family” group decreased as constraint within the duocentric network increased for husbands (90% reduction in odds) and wives (93% reduction). Husbands in the “Family” group had significantly lower constraint compared to husbands in each of the other groups (rrr = .87–.90, *p* < .05). Couples in the “Extreme Disconnection” group had significantly higher constraint among wives compared to wives in the “Family” (rrr = 1.13, *p* < .001) and “Wife Friend” (rrr = 1.1, *p* < .01). Relationship satisfaction for husbands and wives, separately or with their scores combined into a couple score, was not significantly related to cluster membership. Couple relationship length was also not significantly associated with cluster type.

Most measures of duocentric relationship quality were associated with network type. For each 10% increase in network members with whom couples knew “very well,” the odds of membership in the “Family” group increased by 4% and membership in the “Extreme Disconnection” group decreased by 3%. “Family” couples had significantly higher proportion of alters they knew “very well” than each of the other groups (rrr = 1.03–1.04, *p* < .05). Similarly, for each 10% increase in network members with whom couples rated their relationship as “good,” the odds of membership in the “Family” group increased by 2% and membership in the “Extreme Disconnection” group decreased by 2%. The proportion of “good” relationships was significantly higher among those in the “Family” group compared to those in the “Extreme Disconnection” group (rrr = 1.03, *p* = .02).

Increases in the proportion of alters who provided tangible support were associated with lower odds of belonging to the “Extreme Disconnection” group by 4% and this group had significantly lower duocentric tangible support than each of the other groups (rrr = .96–.97, *p* < .05). For each 10% increase in network members who provided emotional support, the odds of membership in the “Shared Friends” group increased by 3% and membership in the “Extreme Disconnection” group decreased by 2%. Couples in the “Shared Friends” group received emotional support from a significantly higher proportion of their networks than each of the other groups (rrr = 1.03–1.04, *p* < .05). For each 10% increase in network members who were rated as approving the marriage, the odds of membership in the “Family” group increased by 2% and membership in the “Extreme Disconnection” group decreased by 3%. Proportion of network approval was significantly lower among those in the “Extreme Disconnection” group relative to both the “Family” (rrr = .95, *p* < .001) and “Shared Friend” (rrr = .92, *p* = .01) groups. Neither of the measures of duocentric network frequency of contact (face to face or virtual) were significantly associated at the 95% confidence level with the odds of group membership. Couples in the “Family” group had significantly higher face-to-face contact with network members than those in the “Shared Friends” group (rrr = 1.01, *p* < .05).

## 4. Discussion

The results presented in this paper represent the first classification of newlywed couple duocentric networks into conjugal types. Moreover, this study is one of the few to analyze duocentric network data for intimate couples and also one of the few to classify couple network data into conjugal networks. As expected, we were able to identify meaningful, qualitatively different types of newlywed couple networks, and we confirmed our expectation that the relative numbers of types of network members, in particular family and friends, would be important determinants of group membership. Also, as we expected, network contacts who were primarily associated with one spouse or were shared between spouses were also a key factor in determining network type. These findings echo the findings of previous studies that have identified conjugal network types determined by relative amounts of family and friends that are either shared or mainly associated with either the husband or wife (Fiori et al., 2017; Stein et al., 1992; Widmer et al., 2004). A detailed comparison of the specific network types generated in the analyses presented here with previous studies has limited value because of methodological differences with previous studies. Stein et al. (1992) is the only other study to analyze duocentric data, but did not analyze any measures of network structure and did not test for associations with network quality and did not analyze the type of structural measures that could be used to generate constraint measures. The conjugal types identified by Fiori et al. (2017) and Widmer et al. (2004) were based on measures that simultaneously assessed network composition and relationship quality with global assessments that cannot be disaggregated into separate measures.

Structural measures of bonding and bridging capital, such as overall and subgroup density and components, were primary drivers of group type, which also matched our expectations. Group types fell into two categories: those with relatively high interconnections among network members and those with low connections. Within these broad categories, density of ties also explained differences among subgroups. The duocentric approach enabled precise comparisons of names listed by husbands and wives to determine which network members were named by both spouses. This measure of shared nominations was a key driver of group classification, with each of the family-centered groups and the “Shared Friend” groups having relatively high amounts of shared nominations.

We also found support for our expectation that group types would differ based on the quality of relationship that couples had with their network members. Couples in one of the family groups had significantly higher proportions of network members they knew very well, had good relationships with, and were supportive of their relationships. Couples in the “Shared Friend” group received significantly more emotional support than other types of couples while couples in the “Extreme Disconnection” group were low on network relationship quality on nearly every measure. In addition to self-reported relationship quality, examining spousal constraint provided insight into social benefits of different types of conjugal networks. We found that husbands and wives in family-centered networks have lower constraint and, therefore, higher bridging social capital, than other types of conjugal networks. This finding is in contrast with the common assumption that networks that are kin-centered are likely to be high in bonding capital and low in bridging capital. Couples early in their relationships are combining their separate spousal personal networks which, separately, may be high in bonding capital with many interconnected family members. Spouses with kin-centric egocentric networks high in bonding capital are able to form duocentric networks that are more structurally novel with many bridging opportunities than their separate egocentric networks.

In contrast, wives had higher constraint in the “Extreme Disconnection” group compared to other groups. Although low connectivity often produces the structural holes necessary to trigger unique flows of ideas and resources (Burt, 2004), networks with extremely low density have low average degree. Couples in the “Extreme Disconnection” group had significantly fewer ties that they rated as knowing “very well” (the edge definition for the constraint calculation). Therefore, spouses in this group, especially wives, had fewer direct strong ties and, therefore, lower social capital than couples in other groups. These tests for association between couple network relationship quality and conjugal network types are unique among studies that have classified network couples.

Beyond these tests of association between network relationship measures and network types, we found a mixture of results for associations between network types and characteristics of couples and their relationships. Couples with older husbands were more likely to be in the “Extreme Disconnection” group. As husbands’ and wives’ ages were significantly correlated for the study sample (*r* = .76, *p* < .001), this suggests that older couples start out their first marriages with less integrated social networks than younger couples. Although older couples on the whole may be likely to have networks with greater connectivity when considering the “withdrawal hypothesis” (Kalmijn, 2003), the couples in this study are all in the early stages of their marriages, which is presumably in the early stages of the social withdrawal process. Although our sampling strategy was chosen to oversample couples experiencing low incomes, the conjugal types were associated with other key determinants of SES, such as education and race/ethnicity. Higher education for both spouses and White ethnicity predicted classification into the “Shared Friend” group and couples that were neither White nor Black were more likely to fall into the “Extreme Disconnection” group, relative to Latinos. As the majority of these couples were of mixed race/ethnicity, this may indicate that couples from different race/ethnic backgrounds have barriers to developing integrated duocentric networks. These findings suggest a need for further research to understand the role of cultural and socioeconomic factors on the formation of couple networks in their relationships prior to marriage and more research on diverse samples (Karney, 2021).

Surprisingly, unlike other studies of couple network types, we did not find any association between duocentric network type and relationship satisfaction. The most likely contributor to this lack of association is homogeneity in marriage length among couples sampled for this study. As the data analyzed for this study are from the first wave of data collection for a study of couples who were recently married, relationship satisfaction is likely at the highest point for these couples and is likely to decline over the initial years of their marriage (see Karney & Bradbury, 2020). It is possible that, although network type does not predict relationship satisfaction in the immediate months after a marriage is formalized, it may predict declines in relationship satisfaction over time. Also, as the “withdrawal hypothesis” predicts, couple networks are expected to evolve over time. This change process, whereby duocentric networks become either more or less connected over time, may be associated with changes in marital satisfaction. We are unable to address this hypothesis in this study because the data is cross-sectional and, based on our findings, associations with network change and relationship trajectories can only be speculated. Measures of network change over time are required to test theories related to networks and relationship outcomes. A priority for future research is to test this association directly through repeated measurements of duocentric network characteristics in newlywed couples over the course of their relationship.

Although the current study provides an essential first step toward understanding the role that a shared couple social networks play in the lives of married couples in the early stages of marriage, it is not without limitations. First, like all studies of personal networks, our network measures are based on cognitions about social relationships and may not always correspond with an objective network. As we acknowledge this limitation, we also note that it is mitigated by our duocentric approach, which combines separate personal network assessments into one network that extends beyond the perception of either spouse. Also, perceptions of network connections may not represent objective reality, even though they have been found to predict relationship outcomes (Fiori et al., 2020). Second, we used a single name generator to generate lists of network contacts from spouses. Some have argued against single name generators for personal network interviews (Neal & Neal, 2017). To maximize the value of this approach, we coupled this name generator with a standardized minimum number of alters, which better enables comparisons of measures of personal network structure than multiple name generators that may produce networks of different sizes (Maya-Jariego, 2018, 2021). As identifying types of couple networks was the primary aim of this study, standardizing the number of alters was an essential design choice (Maya-Jariego et al., 2020).

Another limitation to our findings is that the couples sampled for this study come from a limited geographic region and our sampling procedures were chosen to identify mixed-gender newlywed couples living with low incomes. Therefore, our findings may not generalize to all couples. However, as couples with higher education and higher incomes represent the vast majority of participants in research on marriage and intimate relationships (Karney, 2021), our findings contribute important insights into relationships from under-studied groups. For example, compared to other studies of conjugal networks, our study is the only study to include a sample of couples representing a range of ethnicities and the only study to include a sizable proportion of Latino couples (44.9%).

The cluster analysis method we used to generate couple network types also has some limitations. While the choice of cluster analysis is a strength of the study because it matches our goal of classifying duocentric couple networks, cluster analysis is an inductive, descriptive method for identifying patterns in data and is sensitive to the variables chosen as inputs (Bartholomew et al., 2002). Other studies of couple networks that conduct cluster analyses with different variables may produce different clusters. The cluster analysis algorithm will identify types regardless of data used as an input, even randomly distributed data (Henry et al., 2005); generating “meaningful” clusters with variables selected based on theory is therefore essential to a successful application of cluster analysis. We described how we selected variables informed by social capital theory and previous empirical research and described how these informed our interpretation of clusters. Other studies of couple networks with different theoretical expectations may choose different variables and, therefore, produce different results.

Finally, our focus on mixed-gender newlywed couples produced a homogenous sample of recently married male husbands and female wives. It is unknown how well our couple network typology would describe the networks of other types of couples, such as unmarried intimate partners, those in established marriages, or same-sex couples. It is possible that same-sex couples, who often cultivate “families of choice” to counteract qualified familial acceptance of their relationships (Green & Mitchell, 2002), would have conjugal networks that are very different than mixed-gender newlywed couples. Comparison of data collected from different types of couples to those in this study would generate insight into drivers of conjugal network types.

## 5. Conclusion

Although decades of research on intimate relationships have stressed the importance of social environments for providing couples with social capital, only rarely have these environments been measured precisely. Doing so requires recognition that each couple is the center of their own shared social network and that testing hypotheses about how this network impacts couple outcomes, such as their well-being and the continuation or dissolution of the relationship, requires an appropriate operationalization of that shared network and precise measures of bonding and bridging capital. We have argued that a duocentric approach, which combines elements of personal network data collection, cognitive social structures, and dyadic data analysis, is an appropriate framework for measuring the shared social environment of couples. Using this approach, we identified five distinct types of newlywed couple networks with different configurations of bonding and bridging capital. These network types were associated with varying types of benefits or supports. Further studies of duocentric networks, in particular comparing duocentric networks of different types of couples and testing for associations between the trajectories of duocentric networks and relationship outcomes, can make a significant contribution to advancing both relationship science and personal network research.

## Supplementary Material

1

## Figures and Tables

**Table 1.** Demographic and relationship of couples (*N* = 207)

Characteristic type	Mean (SD)/*N* (%)
Age in years (mean, SD)	
Husband	29.29 (7.28)
Wife	27.64 (6.90)
Race/Ethnicity of couple (*N*, %)	
Both spouses Latino/Hispanic	93 (44.9%)
Both spouses African American	62 (29.9%)
Mixed spouse Race/Ethnicity	36 (17.4%)
Both spouses White	13 (6.3%)
Both spouses Asian/PI	3 (1.5%)
Education in years of school completed (mean, SD)	
Husband	13.74 (3.43)
Wife	14.44 (3.29)
Relationship length in months (mean, SD)	4.82 (4.51)
Total children	2.68 (1.90)
Family income ($1,000/year)	46.21 (34.90)
Relationship satisfaction (mean, SD)	
Husband	43.30 (7.84)
Wife	42.58 (8.59)
Couple score	44.42 (6.44)

**Table 2.** Duocentric network structure and composition characteristics (*N* = 207)

Characteristic type	Mean (SD)
*Criterion cluster measures*	
Network structure	
Density	.20 (.11)
Components	6.73 (6.05)
Overall network composition (proportion)	
Husband family	.19 (.12)
Wife family	.20 (.10)
Husband friend	.18 (.11)
Wife friend	.15 (.08)
Network overlap (proportion)	
Shared friends	.01 (.03)
Both nominated	.12 (.09)
Subgroup density	
Density among husband-nominated alters	.38 (.22)
Density among wife-nominated alters	.35 (.20)
*Duocentered constraint*	
Husband constraint	.14 (.05)
Wife constraint	.14 (.06)
*Duocentric relationship quality*	
Familiarity with alter	
Know very well	.63 (.19)
Know pretty well	.34 (.17)
Know not well	.09 (.11)
Relationship quality	
Relationship good	.81 (.15)
Relationship neutral	.21 (.16)
Relationship bad	.02 (.03)
Support	
Tangible support received	.24 (.17)
Emotional support received	.24 (.18)
Approval of marriage	
Approve	.87 (.18)
Disapprove	.04 (.10)
No opinion	.08 (.16)
Frequency of contact	
Face to face (mean days per year, SD)	91.27 (42.56)
Virtual contact (mean days per year, SD)	90.58 (42.99)

**Table 3.** Within-cluster means and *t*-scores of 5-cluster solution criterion variables and tests of significant differences between clusters

	Cluster 1 (*n* = 46) “Wife Friend”		Cluster 2 (*n* = 60) “Extreme Disconnection”		Cluster 3 (*n* = 27) “Shared Friends”		Cluster 4 (*n* = 60) “Wife Family”		Cluster 5 (*n* = 14) “Husband Family”		ANOVA		
						
Criterion network measure	Mean	*t*-score*	Mean	*t*-score*	Mean	*t*-score*	Mean	*t*-score*	Mean	*t*-score*	η2	*F*-value	*p*-value
Density	.20	100	.11	91	.24	103	.24	103	.40	119	.53	56.41	<.001

Components	7.15	101	12.13	109	3.45	95	3.37	95	2.71	93	.39	32.18	<.001

Husband family	.16	98	.14	96	.23	103	.16	103	.39	117	.29	20.66	<.001

Wife family	.15	95	.19	98	.29	108	.14	108	.22	101	.34	26.09	<.001

Husband friend	.20	101	.21	103	.13	96	.24	96	.07	91	.18	11.01	<.001

Wife friend	.23	110	.14	99	.10	94	.14	94	.11	96	.32	23.65	<.001

Shared friends	.01	98	.00	97	.08	97	.00	97	.00	96	.60	76.43	<.001

Shared nominations	.08	95	.07	93	.17	105	.22	105	.16	104	.44	40.17	<.001

Husband nom. alter density	.45	103	.21	92	.38	100	.41	100	.77	118	.41	34.59	<.001

Wife nom. alter density	.30	97	.24	94	.44	104	.24	104	.55	110	.25	16.65	<.001

* *t*-scores based on mean of 100 and standard deviation of 10. Scores close to 100 represent clusters that have similar means to the full sample. Scores below 100 represent clusters with means below the overall average and scores above 100 represent clusters with means above the overall average.

**Table 4.** Exploratory logistic and multinomial regression predicting membership in one of 5 clusters (vs. non-membership/other clusters) and significant differences between cluster groups

Variables	“Wife Friend”^1^ OR (95% CI)	“Extreme Disconnection”^2^ OR (95% CI)	“Shared Friends”^3^ OR (95% CI)	“Family”^4^ OR (95% CI)
*Couple demographics*			
Husband age	1.00 (0.95, 1.04)	1.0S (1.01,1.09)**^3,4^	.95 (0.88,1.01)^2^	.98 (0.94,1.02)^2^
Wife age	1.02 (0.97,1.07)	1.03 (0.98, 1.07)	.96 (0.89,1.02)	.98 (0.93,1.02)
Husband education level	.81 (0.57,1.13)^3^	1.04 (0.77,1.41)^3^	2.91 (1.83, 4.90)***^1,3,4^	.69 (0.51, 0.93)**^3^
Wife education level	.78 (0.S4, 1.10)^3^	.98 (0.72, 1.3S)^3^	2.13 (1.3S, 3.48)***^1,2,4^	.86 (0.64,1.16)^3^
Race/Ethnicity (Latino reference group)			
Black: Latino	1.49 (0.71, 3.13)	.87 (0.41,1.81)	.51 (0.14, 1.S9)	1.02 (0.53,1.97)
White: Latino	1.10 (0.23, 3.99)	.23 (0.01, 1.24)^3^	8.70 (2.48, 31.97)***^2,4^	.28 (0.04,1.10)^3^
Other: Latino	.54 (0.17,1.46)	2.58 (1.19, 5.67)**^4^	1.10 (0.33, 3.26)	.52 (0.22,1.17)^2^
*Couple relationship characteristics*			
Husband relationship satisfaction	.98 (0.95, 1.03)	.98 (0.95, 1.02)	1.03 (0.97,1.10)	1.02 (0.98,1.06)
Wife relationship satisfaction	.99 (0.95, 1.03)	.99 (0.96, 1.02)	1.03 (0.98,1.10)	1.01 (0.97, 1.04)
Relationship satisfaction couple score	.98 (0.94, 1.03)	.98 (0.94, 1.02)	1.03 (0.97,1.12)	1.02 (0.98, 1.07)
Relationship length	.94 (0.85, 1.02)	1.03 (0.97, 1.10)	1.02 (0.93,1.11)	.99 (0.93, 1.06)
*Duocentered constraint*			
Husband constraint	1.02 (0.95,1.09)^4^	1.03 (0.97,1.10)^4^	1.06 (0.98,1.14)^4^	.90 (0.82,0.97)**^1,2,3^
Wife constraint	.96 (0.88,1.02)^2^	1.11 (1.04,1.19)***^1,4^	.99 (0.90,1.05)	.93 (0.85, 0.99)**^2^
*Duocentered relationship quality*			
^†^Proportion know very well	1.00 (0.98,1.02)^4^	.97 (0.96, 0.99)***^4^	.99 (0.97,1.01)^4^	1.04 (1.02,1.05)***^1,2,3^
Proportion good relationship	.99 (0.97,1.01)	.98 (0.96,1.00)**^4^	1.02 (0.99,1.05)	1.02 (1.00,1.04)**^2^
Proportion tangible support received	1.00 (0.98,1.02)^2^	.96 (0.94, 0.99)***^1,3,4^	1.02 (1.00,1.04)^2^	1.02 (1.00,1.03)*^2^
Proportion emotional support received	1.00 (0.98,1.02)^3^	.98 (0.96,1.00)**^3^	1.03 (1.01,1.05)***^1,2,4^	1.00 (0.98,1.02)^3^
Proportion approve of marriage	1.00 (0.98,1.02)	.97 (0.96, 0.99)***^3,4^	1.03 (1.00,1.08)*^2^	1.02 (1.00,1.04)**^2^
Days face-to-face contact	1.00 (0.99,1.01)	1.00 (0.99,1.00)	.99 (0.98,1.00)*^4^	1.01 (1.00,1.01)*^3^
Days virtual contact	1.00 (1.00,1.01)	.99 (0.98,1.00)*	1.00 (0.99,1.01)	1.00 (1.00,1.01)

* Bivariate logistical regression *p*-value ≤.10.

** Bivariate logistical regression *p*-value ≤.05.

*** Bivariate logistical regression *p*-value ≤.01.

1 Multinomial model significant with “Wife Friend” as the reference group.

2 Multinomial model significant with “Extreme Disconnection” as the reference group.

3 Multinomial model significant with “Shared Friend” as the reference group.

4 Multinomial model significant with “Family” as the reference group.

† Proportions converted to proportion deciles by multiplying by 10 to make results more interpretable.
