# Supplementary material for: Typologies of duocentric networks among low-income newlywed couples
Source: Netw Sci (Camb Univ Press). Author manuscript; Available in PMC 2024 Jun 1. (PMC10783164; doi:10.1017/nws.2023.16)
Supplement: 1 [file NIHMS1918476-supplement-1.docx]

**APPENDIX**

**Appendix A: Methodological Notes**

**Q1:** How does requiring participants to name 25 people and referring to it as their network shape interpretations? Although I recognize that such an approach is necessary given Kennedy et al.’s (2015) work suggesting that number is needed to construct duocentric networks, does it artificially inflate the size of the networks that spouses actually rely on in their day-to-day lives?

**Response**: Our approach to generating 25 names was to ask for the spouse first and then 24 additional people without prompting for any specific type of person, such as those who they actually rely on in their day-to day lives. This approach of asking respondents to name a “large” number of people with a generic name generator (non-specific to a type of person) leaves it up to the respondent to decide who to name. Once the list is generated, we then ask the participant to classify the people as supportive or not, family, friends, etc. The measures of types of network members (e.g. those who provide support) is based on the name interpreter questions, not the number of names generated from the name generators alone. We agree that if we asked respondents to name a specific type of 25 people, for example those that they relied on for support, this would inflate the size of the support network. Because this study is focused on both strong ties (bonding capital) and weak ties (bridging capital), we prioritized generating a list of names that was large enough to generate both strong and weak ties and then produce counts/proportions based on the answers to name interpreter questions.

We agree that requesting a fixed number of alters with a single name generator is a methodological choice that has advantages and disadvantages, like any other methodological choice. We describe some of these limits in the discussion section. To justify our belief that the advantages outweigh the limitations, we cite the following papers that have argued for the benefits of generating a fixed number of alters, in particular for generating data for a cluster analysis:

Maya-Jariego, I. (2021). Building a structural typology of personal networks: Individual differences in the cohesion of interpersonal environment. Social Networks, 64, 173-180. doi:10.1016/j.socnet.2020.09.006

Maya-Jariego, I. (2018). Why Name Generators with a Fixed Number of Alters may be a Pragmatic Option for Personal Network Analysis. American Journal of Community Psychology, 62(1-2), 233-238. doi:10.1002/ajcp.12271

These papers note that structural measures are highly sensitive to network size and standardizing network size enables comparisons. Our choice to standardize the network sizes was also informed by a concern that an unbounded list would be biased towards being too small to accurately represent the people in the respondents’ lives. This problem happens when some respondents and interviewers learn that the fewer names elicited in a name generator, the fewer questions they will have to ask/answer if the list of names is smaller. There have been a number of empirical studies of this learning effect. For example:

Marsden, P. V. (2003). Interviewer effects in measuring network size using a single name generator. Social Networks, 25(1), 1-16. doi:http://dx.doi.org/10.1016/S0378-8733(02)00009-6

Valente, T. W., Dougherty, L., & Stammer, E. (2017). Response Bias over Time: Interviewer Learning and Missing Data in Egocentric Network Surveys. Field Methods, 29(4), 1525822X17703718. doi:10.1177/1525822X17703718

We believe that asking for 25 names and then classifying them according to closeness, support, etc. after the list has been generated helps to correct against this potential bias. If a sub-set of respondents/interviewers generated small networks because they wanted to end the interview sooner, the cluster analysis would produce a set of groups biased towards network size that did not necessarily reflect the true size of respondents’ networks.

We also believe that defining an egocentric network always requires choices about how to operationalize the boundary. Not every egocentric study has the same research goals and each will operationalize the egocentric boundary in different ways. We believe that setting a network definition of a non-specific 24 names + 1 spouse is reasonable when considering the true size of personal networks is likely much larger than 25. There have been a number of empirical and simulation studies have attempted to quantify personal network size in human beings. For example:

Hill, R. A., & Dunbar, R. I. M. (2003). Social network size in humans. Human Nature-an Interdisciplinary Biosocial Perspective, 14(1), 53-72. doi:10.1007/s12110-003-1016-y

McCormick, T. H., Salganik, M. J., & Zheng, T. (2010). How Many People Do You Know?: Efficiently Estimating Personal Network Size. Journal of the American Statistical Association, 105(489), 59-70. doi:10.1198/jasa.2009.ap08518

Killworth, P. D., McCarty, C., Johnsen, E. C., Bernard, H. R., & Shelley, G. A. (2006). Investigating the variation of personal network size under unknown error conditions. Sociological Methods & Research, 35(1), 84-112. doi:10.1177/0049124106289160

These studies have produced a range of estimates, but they are all well above 25 (ranging from hundreds to thousands). Based on the findings of Kennedy et al., we chose 25 because it was likely to include both strong and weak ties for newlywed couples.

Kennedy, D. P., Jackson, G. L., Green, H. D., Bradbury, T. N., & Karney, B. R. (2015). The Analysis of Duocentric Social Networks: A Primer. Journal of Marriage and Family, 77(1), 295-311. doi:10.1111/jomf.12151

Finally, although we calculated the overall network size for each duocentric network, we did not include this variable as an individual measure for determining the clusters. This measure was used to standardize the proportions of family members, friends, etc. because the duocentric network size changed based on the number of alters who were named by both spouses.

**Q2**: Regarding the relationship quality with alters, was neutral conceptualized as being neither positive nor negative or could participants use neutral if the relationship was ambivalent (e.g., both positive and negative)? What were the instructions regarding this designation?

Response: “Neutral” was not defined for respondents. They may have considered their relationships with alters both positive and negative or neither positive nor negative. They were not provided with any other instructions other than the response options.

**Q3**: How many regression models were run in total? Did you consider paring down the number of models?

**Response:** We considered reducing the number of models to present. However, we were unable to identify models appropriate for removal from the analysis.

We ran 18 different bivariate models with the groups as the dependent variables. There were 4 complimentary sets of logistic regression models (one for each group as the reference). The multinomial logistic regression models included the same independent variables with pairs of comparisons among the groups as the dependent variable. Therefore, although separately there were a large number of models (18 x 4 + 18 = 90), there were essentially 18 tests of association between independent variables and the same dependent variable using different types of analysis and operationalizations of the groups. Eight of these tests were on the same independent variable measured for husbands and wives separately.

We considered removing the companion multi-nominal models or the logistic regression models, but we believe that presenting these models together helps to show which groups had the strongest associations with the independent variables (logistic regression) and which groups were the most different from each other (multinomial regression).

We also considered removing the relationship satisfaction analysis. None of these variables were associated with the categorization; therefore, removing these variables would reduce the number of analyses and results to describe. However, we were reluctant to remove these null findings because other studies have found associations between relationship satisfaction and types of couple networks.

**Q4**: Table 4 tests how measures of duocentric constraints and duocentric relationship quality are related to the network types. These measures overlap with the measures used in the factor analysis to derive the five clusters, i.e. alter density (part of cluster analyses) overlaps with duocentered constraints and the proportion of "know very well". Is this problematic and if not, why not?

**Response:** Constraint and density are related but constraint is a positional, node level measure and includes sensitivity to structural aspects for the direct and indirect connections to the spouse nodes within the duocentric network, whereas density is a measure at the network level.

In contrast, each of the measures included as inputs into the cluster analyses are based on duocentric networks that exclude the spouses. Duocentric density was calculated on the networks formed without the spouses included as nodes (similar to components). We added clarifying text to make this more clear. The cluster analysis was based on networks that included relationship ties that were defined as knowing "pretty" well or "very" well whereas the constraint measure was based on the network formed with "very" well ties only.

Together, these factors reduce the overlap between the measures. To further explore how related these measures are, we looked at the correlation between the measures and found that it was low. The correlation between density and constraint was -.205 for husbands and -.228 for wives.

**Appendix B: Cluster Analysis Diagnostics**


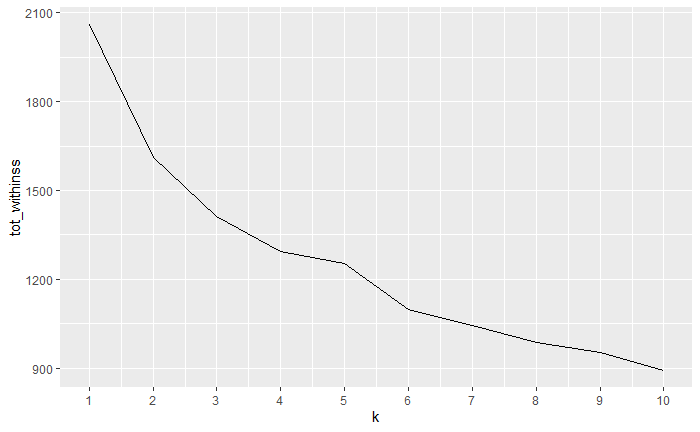


**Supplementary Figure 1** Within Cluster Sum of Squares Elbow Plot. The figure shows that the 5-cluster solution produced the most noticeable bend in the within-cluster sum of squares elbow plot with another noticeable bend at 2-clusters.


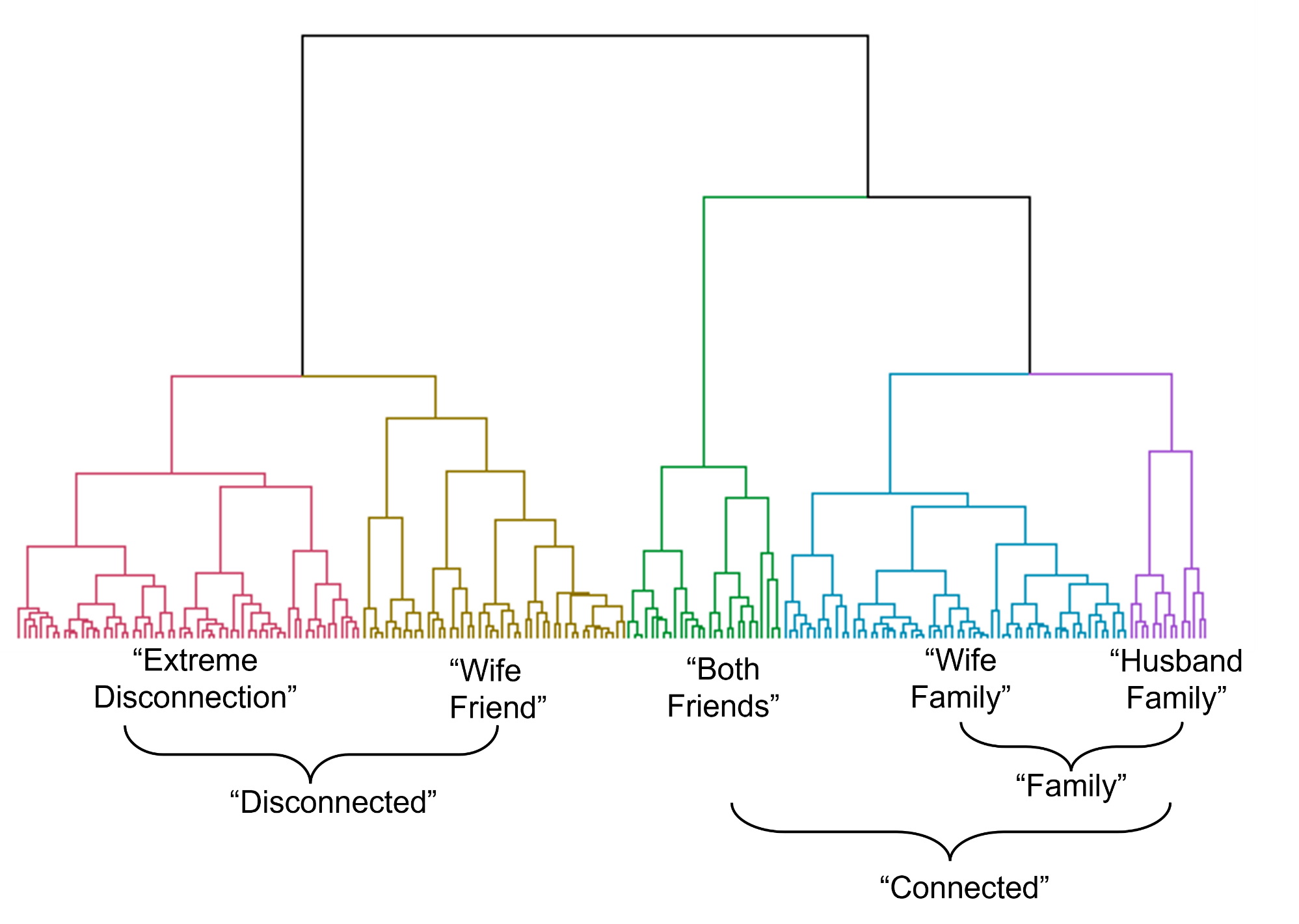


**Supplementary Figure 2** Dendogram of 2 and 5 Cluster Solutions


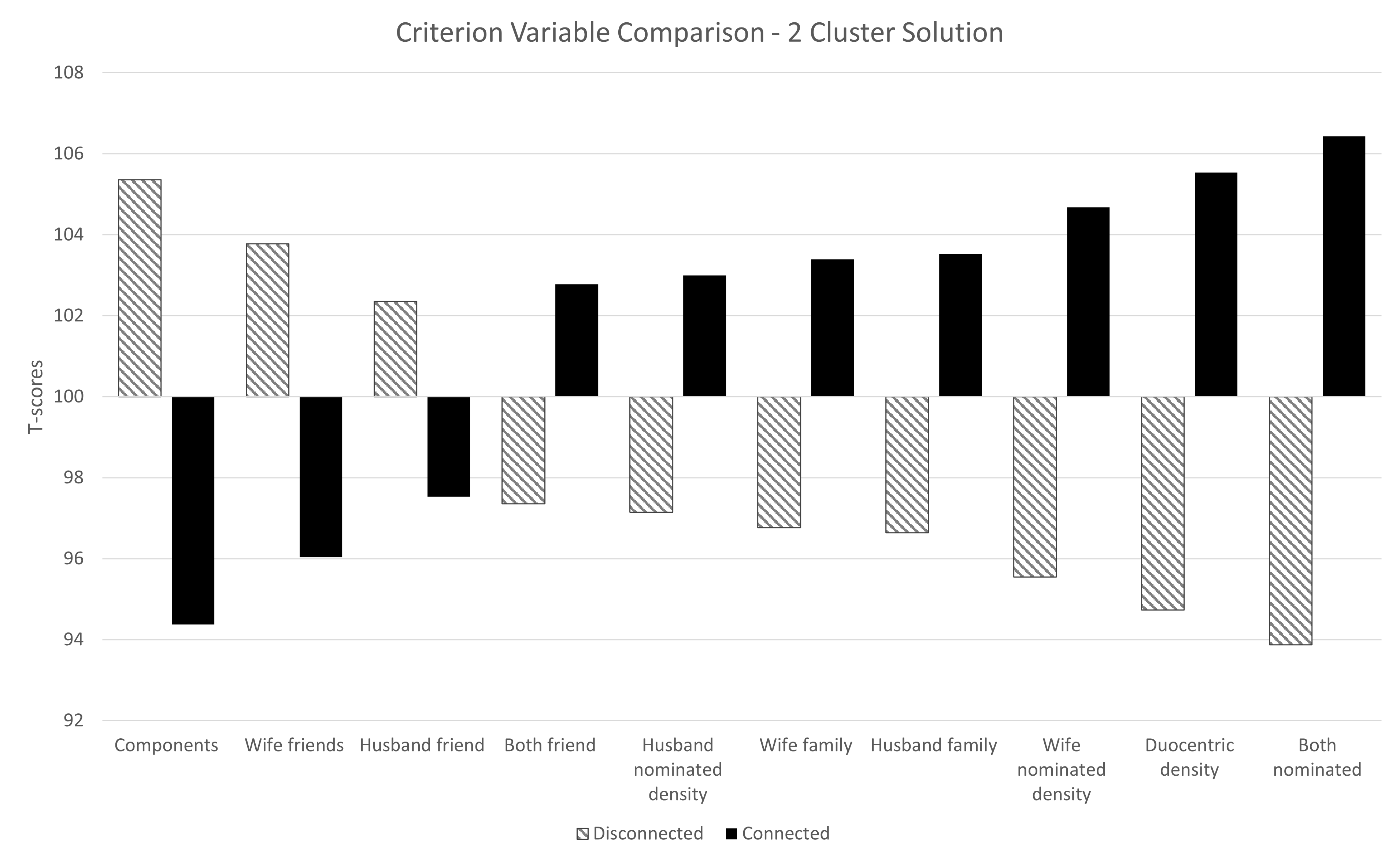


**Supplementary Figure 3** Standardized mean scores for criterion variables by network type for 2 cluster solution (M = 100, SD = 10). Raw measures of criterion variables have been converted to t-scores with mean = 100 and standard deviation = 10 in order to standardize the height of the bars to facilitate visual comparison among the variables.
